# Assessing Health Students' Attitudes and Usage of ChatGPT in Jordan: Validation Study

**DOI:** 10.2196/48254

**Published:** 2023-09-05

**Authors:** Malik Sallam, Nesreen A Salim, Muna Barakat, Kholoud Al-Mahzoum, Ala'a B Al-Tammemi, Diana Malaeb, Rabih Hallit, Souheil Hallit

**Affiliations:** 1 Department of Pathology, Microbiology and Forensic Medicine School of Medicine The University of Jordan Amman Jordan; 2 Department of Clinical Laboratories and Forensic Medicine Jordan University Hospital Amman Jordan; 3 Prosthodontic Department School of Dentistry The University of Jordan Amman Jordan; 4 Prosthodontic Department Jordan University Hospital Amman Jordan; 5 Department of Clinical Pharmacy and Therapeutics Faculty of Pharmacy Applied Science Private University Amman Jordan; 6 Middle East University Research Unit Middle East University Amman Jordan; 7 Migration Health Division International Organization for Migration The United Nations Migration Agency Amman Jordan; 8 College of Pharmacy Gulf Medical University Ajman United Arab Emirates; 9 School of Medicine and Medical Sciences Holy Spirit University of Kaslik Jounieh Lebanon; 10 Department of Infectious Disease Bellevue Medical Center Mansourieh Lebanon; 11 Department of Infectious Disease Notre Dame des Secours University Hospital Center Byblos Lebanon; 12 Research Department Psychiatric Hospital of the Cross Jal Eddib Lebanon

**Keywords:** artificial intelligence, machine learning, education, technology, healthcare, survey, opinion, knowledge, practices, KAP

## Abstract

**Background:**

ChatGPT is a conversational large language model that has the potential to revolutionize knowledge acquisition. However, the impact of this technology on the quality of education is still unknown considering the risks and concerns surrounding ChatGPT use. Therefore, it is necessary to assess the usability and acceptability of this promising tool. As an innovative technology, the intention to use ChatGPT can be studied in the context of the technology acceptance model (TAM).

**Objective:**

This study aimed to develop and validate a TAM-based survey instrument called TAME-ChatGPT (Technology Acceptance Model Edited to Assess ChatGPT Adoption) that could be employed to examine the successful integration and use of ChatGPT in health care education.

**Methods:**

The survey tool was created based on the TAM framework. It comprised 13 items for participants who heard of ChatGPT but did not use it and 23 items for participants who used ChatGPT. Using a convenient sampling approach, the survey link was circulated electronically among university students between February and March 2023. Exploratory factor analysis (EFA) was used to assess the construct validity of the survey instrument.

**Results:**

The final sample comprised 458 respondents, the majority among them undergraduate students (n=442, 96.5%). Only 109 (23.8%) respondents had heard of ChatGPT prior to participation and only 55 (11.3%) self-reported ChatGPT use before the study. EFA analysis on the attitude and usage scales showed significant Bartlett tests of sphericity scores (*P*<.001) and adequate Kaiser-Meyer-Olkin measures (0.823 for the attitude scale and 0.702 for the usage scale), confirming the factorability of the correlation matrices. The EFA showed that 3 constructs explained a cumulative total of 69.3% variance in the attitude scale, and these subscales represented perceived risks, attitude to technology/social influence, and anxiety. For the ChatGPT usage scale, EFA showed that 4 constructs explained a cumulative total of 72% variance in the data and comprised the perceived usefulness, perceived risks, perceived ease of use, and behavior/cognitive factors. All the ChatGPT attitude and usage subscales showed good reliability with Cronbach α values >.78 for all the deduced subscales.

**Conclusions:**

The TAME-ChatGPT demonstrated good reliability, validity, and usefulness in assessing health care students’ attitudes toward ChatGPT. The findings highlighted the importance of considering risk perceptions, usefulness, ease of use, attitudes toward technology, and behavioral factors when adopting ChatGPT as a tool in health care education. This information can aid the stakeholders in creating strategies to support the optimal and ethical use of ChatGPT and to identify the potential challenges hindering its successful implementation. Future research is recommended to guide the effective adoption of ChatGPT in health care education.

## Introduction

Health care education has a rich history marked by notable revolutionary milestones [1-8]. The latest potential milestone could be the incorporation of artificial intelligence (AI) and machine learning (ML) into this educational domain with the capacity to bring about promising transformative changes [9-12]. The past decade has witnessed significant advancements in the application of AI and ML to health care education and practice [13-16].

Advanced AI-based tools, such as Generative Pretrained Transformer (GPT)–based tools developed by OpenAI, have the potential to significantly impact health care education [17]. These tools implement deep neural networks for generating human-like texts in various languages [17]. The high accuracy and promising potential of these tools can advance health care education [9,18]. The publicly available and user-friendly ChatGPT from OpenAI exemplifies the widespread attention and scrutiny received in academia and among health professionals [9,17,19-21].

The successful implementation of novel technologies is influenced by a range of factors, including technical, social, cultural, and psychological aspects that shape attitudes and behaviors toward the technology [22-24]. To achieve this goal, various frameworks have been developed, such as the technology acceptance model (TAM) [25,26] and the Unified Theory of Acceptance and Use of Technology 2 (UTAUT2) [27-29], among others [30,31]. These models help elucidate the interplay of complex factors that shape the acceptance and usage of novel technologies [32]. The popularity of TAM stems from its valid and straightforward framework, enabling the study of factors that motivate the adoption of technological innovations [32,33].

In examining the acceptance and usage of novel technology, the TAM framework utilizes constructs that assess the perceived usefulness, ease of use, risks, anxiety, attitude toward the technology, social influence, and cognitive and behavioral factors [25,26].

Since its public release in November 2022, ChatGPT has evoked both enthusiasm and concerns [34-37]. The same controversy has soared in the context health care research, education, and practice settings [9]. The utility of ChatGPT in health care education has been reviewed recently [9]. Its cited benefits included enhancing personalized learning experiences, potentially enhancing communication skills, and increasing students’ engagement in the learning process [9,18,38,39].

However, several valid concerns were raised, including the possibility of generating inaccurate content, along with ethical issues, including the risk of bias, plagiarism, and copyright issues [9,18,40,41]. Understanding the acceptance and use factors among health care students is essential, and the TAM framework offers a comprehensive yet simple approach for this purpose.

The rationale of such a study is justified based on several factors. First, ChatGPT’s novelty and potential in health care education necessitate an understanding of its acceptance and the factors influencing it. Second, ChatGPT’s transformative potential in self-learning, feedback, and problem-solving warrants investigation for effective integration. Third, exploring health care students’ attitudes sheds light on technology readiness and benefits. Finally, understanding student attitudes aids in addressing ethical concerns for responsible utilization of ChatGPT in health care settings.

Therefore, this study aimed to establish and test a TAM-based construct for understanding the acceptance and use of ChatGPT, a novel technology, among university students in health care disciplines. This study sought to analyze the possible factors that would drive the successful adoption and implementation of ChatGPT as an example of large language models (LLMs) in health care education. Consequently, the survey instrument developed in this study can provide valuable insights into the factors influencing the adoption of this transformative tool.

## Methods

### Inclusion and Exclusion Criteria

Potential study participants were recruited by convenience sampling through the authors’ contacts in Jordan. The survey link was sent through WhatsApp and Facebook groups targeted to students in health schools in the Arab-speaking country. The survey was open from February 28, 2023, and was closed on March 31, 2023. Participation was voluntary and did not involve incentives. The inclusion criteria that were outlined explicitly in the introductory section of the questionnaire before the informed consent item included (1) being 18 years of age or older, (2) being concurrently enrolled in a Jordanian university, and (3) having a very good comprehension of the Arabic language. The exclusion criteria included (1) being younger than 18 years of age, (2) studying in non–health care-related disciplines, (3) having a poor comprehension of the Arabic language.

The minimum sample size was estimated to be 360 participants following the established guidelines for survey validation studies, considering 36 items with 10 participants per item [42-44].

### Ethics Approval

This study was approved by the institutional review board of the School of Pharmacy at the Applied Science Private University (2023-PHA-3), and approval was granted on January 24, 2023. Participation was voluntary and anonymous.

### Construction of the Survey Instrument to Assess the Acceptance and Usage of ChatGPT

The survey instrument development process involved an extensive literature review and expert validation, followed by item development and pilot testing to ensure clarity [25,26,45-49]. Following an internal discussion among the authors with previous experience in survey construction and validation (MS, MB, DM, and SH), the survey tool was created based on the TAM framework. This internal discussion led to the identification of potential domains for inclusion in the final questionnaire: perceived usefulness, ease of use, risks, anxiety, attitude toward the technology, social influence, and cognitive and behavioral factors [25,26].

Herein, we refer to this edited TAM model in the context of ChatGPT adoption as the TAME-ChatGPT (Technology Acceptance Model Edited to Assess ChatGPT Adoption) survey instrument. Face and content validity were assessed by subjective evaluation, with an assessment of the clarity, comprehensiveness, and relevance of the initial items that were adopted. Additionally, any potential biases or issues with the wording of the items (eg, vague wording or complex items) were assessed [50].

Then, forward and backward translations were conducted by 3 authors (MS, NAS, and MB). Afterward, the survey was distributed among 6 participants representing a pilot test, followed by minor language modifications to improve clarity. The construct validity was checked following survey distribution using 13 TAM-based items evaluated among the respondents who heard of ChatGPT before the study. An additional 23 TAM-based items were evaluated among the respondents who used ChatGPT before the study.

The survey was introduced with a full explanation of the aims and a mandatory electronic consent item for the successful completion of the survey. The introductory section explicitly explained the guaranteed participant anonymity and privacy by refraining to request any personal details such as names or emails. This was followed by items to assess age, sex, university (public vs private), nationality (Jordanian vs non-Jordanian), school (health vs scientific vs humanities), and current educational level (undergraduate vs postgraduate). Then, a single item followed (“Have you heard of ChatGPT before the study?”) with a “yes” response required to move into the next item, while the answer of “no” resulted in survey submission. The next item was “Have you used ChatGPT before the study?” with “yes” resulting in the presentation of the full 36 items. An answer of “no” resulted in the presentation of the first 13 TAM items. The complete phrasing of the included items is presented in Table S1 of Multimedia Appendix 1.

Each item was evaluated on a 5-point Likert scale with the following responses: strongly agree scored as 5, agree scored as 4, neutral/no opinion scored as 3, disagree scored as 2, and strongly disagree scored as 1. The scoring was reversed for the items implying a negative attitude toward ChatGPT.

### Statistical Analysis of Evaluation of Factorability for the Correlation Matrix of the Attitude and Usage Scales

The statistical analysis was performed using SPSS software (V22.0; IBM Corp). To explore the factor structure of the TAME-ChatGPT construct comprising a total of 36 items, we conducted an exploratory factor analysis (EFA) using principal component analysis (PCA) as the extraction method and oblimin rotation to determine the correlations between factors. The Kaiser-Meyer-Olkin (KMO) measure of sampling adequacy and the Bartlett test of sphericity were used to assess the suitability of the data for EFA. The internal consistency of the subscales and the TAME-ChatGPT was checked using Cronbach α. The level of statistical significance was set at *P*<.05.

### Descriptive Analysis of Attitudes Toward ChatGPT and Its Usage Based on TAME-ChatGPT

Descriptive statistics included the measures of distribution (mean and median), dispersion (SD), and IQR. For the scale variables, and considering the relatively small sample size, the Shapiro-Wilk test was used to assess the normality of the scale variables.

The associations between categorical variables were assessed using the chi-square (*χ*^2^) test, while the associations between categorical and scale variables were assessed by the Mann-Whitney (M-W) *U* test for nonnormally distributed scale variables. The level of statistical significance was *P*=.05.

## Results

### Study Participants

A total of 480 responses were received over a 1-month period. A total of 9 individuals declined to participate in the study. Moreover, 5 respondents attending humanities schools and 8 science students were excluded. Thus, the final study sample comprised a total of 458 participants.

The study sample had a mean age of 21 (SD 3.3) years and a median age of 20 (IQR 19-22) years. Characteristics of the study sample are shown in Table 1. Out of the 458 participants, only 109 (23.8%) had heard of ChatGPT prior to the study, and only 55 (11.3%) self-reported ChatGPT use before the study.

**Table 1:** Characteristics of the study respondents (N=458).

Categories		Values, n (%)
**Age (years)**		
	18-20 years	251 (54.8)
	>20 years	207 (45.2)
**Sex**		
	Male	143 (31.2)
	Female	315 (68.8)
**Nationality**		
	Jordanian	207 (45.2)
	Non-Jordanian	251 (54.8)
**University**		
	Public	392 (85.6)
	Private	66 (14.4)
**Educational level**		
	Undergraduate	442 (96.5)
	Postgraduate	16 (3.5)
**Have you heard of ChatGPT before this study?**		
	Yes	109 (23.8)
	No	349 (76.2)
**Have you used ChatGPT before this study?^a^**		
	Yes	55 (50.5)
	No	54 (49.5)

^a^The item was assessed only for the participants who heard of ChatGPT before the study (109/458, 23.8%).

### Prior Knowledge and Usage of ChatGPT Among the Study Participants

In the whole study sample, older age, male sex, and postgraduate education were associated with a higher probability of hearing about ChatGPT before the study (Table 2). On the other hand, the differences lacked statistical significance upon comparing the different categories in the tested variables with the probability of ChatGPT usage before the study (Table 2).

**Table 2:** Association between the study variables and previous knowledge or usage of ChatGPT.

Category			Have you heard of ChatGPT before this study?					*P* value, chi-square (*df*)			Have you used ChatGPT before this study?					*P* value, chi-square (*df*)
			Yes, n (%)		No, n (%)					Yes, n (%)			No, n (%)			
**Age (years)**							.001, 12 (1)								.64, 0.2 (1)	
	18-20	44 (17.5)		207 (82.5)					21 (47.7)			23 (52.3)				
	>20	65 (31.4)		142 (68.6)					34 (52.3)			31 (47.7)				
**Sex**							<.001, 47 (1)								.39, 0.7 (1)	
	Male	63 (44.1)		80 (55.9)					34 (54)			29 (46)				
	Female	46 (14.6)		269 (85.4)					21 (45.7)			25 (54.3)				
**Nationality**							.30, 1.1 (1)								.29, 1.1 (1)	
	Jordanian	54 (26.1)		153 (73.9)					30 (55.6)			24 (44.4)				
	Non-Jordanian	55 (21.9)		196 (78.1)					25 (45.5)			30 (54.5)				
**University**							.40, 0.7 (1)								.74, 0.1 (1)	
	Public	96 (24.5)		296 (75.5)					49 (51)			47 (49)				
	Private	13 (19.7)		53 (80.3)					6 (46.2)			7 (53.8)				
**Educational level**						.002, 9.6 (1)								.31, 1 (1)		
	Undergraduate	100 (22.6)		342 (77.4)					49 (49)			51 (51)				
	Postgraduate	9 (56.3)		7 (43.8)					6 (66.7)			3 (33.3)				

### Factorability of the Correlation Matrix of the Attitude Scale

The EFA was conducted on a set of 13 items to identify underlying factors that accounted for the variance in the responses. The sample comprised the participants who heard of ChatGPT before the study (n=109, 23.8%). The Bartlett test of sphericity was significant (*χ*²_78_=779.2) *P*<.001), indicating the factorability of the correlation matrix. The KMO measure of the sampling adequacy was 0.823, indicating that the data were suitable for factor analysis.

The EFA was performed using PCA and oblimin rotation to account for potential correlations between factors. The scree plot showed that the optimal number of factors was 3, which explained a cumulative total of 69.3% of the variance in the data (Figure S1 of Multimedia Appendix 1). The eigenvalues for the 3 factors were 4.695, 3.148, and 1.168, respectively. All 13 items loaded significantly on 1 of the 3 factors, with factor loadings ranging from 0.65 to 0.87 (Table 3).

Based on the original TAM constructs, factor 1 was labeled “perceived risk” and included 5 items. Factor 2 was labeled “technology/social influence” and included 5 items related to attitude toward technology and social influence. Factor 3 was labeled “anxiety” and included 3 items related to anxiety and fear from ChatGPT.

The 3 factors demonstrated good internal consistency, with Cronbach α values of .876, .858, and .827, respectively, indicating that they could be used to measure these constructs in future research.

**Table 3:** Pattern matrix of the principal component analysis showing the 3 inferred factors for the attitude scale.

Component	Factor 1 (Perceived risk)	Factor 2 (Technology/social influence)	Factor 3 (Anxiety)
1. I am concerned about the reliability of the information provided by ChatGPT^a^.	0.743	<0.400	<0.400
2. I am concerned that using ChatGPT would get me accused of plagiarism^a^.	0.873	<0.400	<0.400
3. I am afraid of relying too much on ChatGPT and not developing my critical thinking skills^a^.	<0.400	<0.400	0.839
4. I am concerned about the potential security risks of using ChatGPT^a^	0.652	<0.400	<0.400
5. I am afraid of becoming too dependent on technology like ChatGPT^a^.	<0.400	<0.400	0.869
6. I am afraid that using ChatGPT would result in a lack of originality in my university assignments and duties^a^.	<0.400	<0.400	0.732
7. I am afraid that the use of the ChatGPT would be a violation of academic and university policies^a^.	0.807	<0.400	<0.400
8. I am concerned about the potential privacy risks that might be associated with using ChatGPT^a^.	0.695	<0.400	<0.400
9. I am enthusiastic about using technology such as ChatGPT for learning and research.	<0.400	0.828	<0.400
10. I believe technology such as ChatGPT is an important tool for academic success.	<0.400	0.837	<0.400
11. I think that technology like ChatGPT is attractive and fun to use.	<0.400	0.868	<0.400
12. I am always keen to learn about new technologies like ChatGPT.	<0.400	0.775	<0.400
13. I trust the opinions of my friends or colleagues about using ChatGPT.	<0.400	0.717	<0.400

^a^Items were reversed coded.

### Factorability of the Correlation Matrix of the Usage Scale

The EFA was conducted on a set of 14 items to identify underlying factors that accounted for the variance in the responses. The sample comprised the participants who used ChatGPT before the study (n=55, 11.3%). The Bartlett test of sphericity was significant (*χ*²_91_=427.1; *P*<.001), indicating the factorability of the correlation matrix. The KMO measure of sampling adequacy was 0.702, indicating that the data were suitable for factor analysis.

Similar to the approach used for the attitude scale, the EFA was performed using PCA and oblimin rotation. The scree plot indicated that the optimal number of factors was 4, which explained a cumulative total of 72% of the variance in the data (Figure S2 of Multimedia Appendix 1). The eigenvalues for the 4 factors were 5.296, 1.979, 1.577, and 1.269, respectively. All 14 items loaded significantly on 1 of the 4 factors, with factor loadings ranging from 0.59 to 0.94 (Table 4).

Factor 1 was labeled “perceived usefulness” and included 6 items related to perceived usefulness. Factor 2 was labeled “perceived risk” and included 3 items related to perceived risk. Factor 3 was labeled “perceived ease of use” and included 2 items related to ease of use. Factor 4 was labeled “behavior” and included 3 items related to cognitive and behavioral aspects of ChatGPT use.

The 4 factors demonstrated good internal consistency (Cronbach α values of .885, .718, .824, and .781, respectively) and could be used to measure these constructs in future research.

**Table 4:** Pattern matrix of the principal component analysis showing the 4 inferred factors for the usage scale.

Component	1 (Perceived usefulness)	2 (Perceived risk)	3 (Perceived ease of use)	4 (Behavior)
2. I am concerned that using ChatGPT would get me accused of plagiarism.	<0.400	0.790	<0.400	<0.400
4. I am concerned about the potential security risks of using ChatGPT.	<0.400	0.840	<0.400	<0.400
14. ChatGPT helps me to save time when searching for information.	0.840	<0.400	<0.400	<0.400
16. For me, ChatGPT is a reliable source of accurate information.	0.664	<0.400	<0.400	<0.400
19. I recommend ChatGPT to my colleagues to facilitate their academic duties.	0.840	<0.400	<0.400	<0.400
20. ChatGPT is more useful than other sources of information that I have used previously.	0.585	<0.400	<0.400	<0.400
22. I have used tools or techniques similar to ChatGPT in the past.	<0.400	<0.400	<0.400	0.703
23. I spontaneously find myself using ChatGPT when I need information for my university assignments and duties.	<0.400	<0.400	<0.400	0.852
24. I often use ChatGPT as a source of information in my university assignments and duties.	<0.400	<0.400	<0.400	0.745
26. I think that relying on technology like ChatGPT can disrupt my critical thinking skills.	<0.400	0.756	<0.400	<0.400
27. I appreciate the accuracy and reliability of the information provided by ChatGPT.	0.614	<0.400	<0.400	<0.400
28. I believe that using ChatGPT can save time and effort in my university assignments and duties.	0.937	<0.400	<0.400	<0.400
30. It does not take a long time to learn how to use ChatGPT.	<0.400	<0.400	0.880	<0.400
32. ChatGPT does not require extensive technical knowledge.	<0.400	<0.400	0.869	<0.400

### Descriptive Analysis of the Attitudes Toward ChatGPT Based on TAME-ChatGPT

The 3 TAME-ChatGPT attitude subscales were evaluated at first. The possible range of the perceived risks subscale was between 5 and 25, with higher values indicating low perceived ChatGPT risks due to reverse coding of these items and a score of 15 indicating a neutral attitude toward ChatGPT.

Among the participants who have heard of ChatGPT before the study (n=109, 23.9%), the mean perceived risks score was 12.5 (SD 4.8), indicating a general agreement with the items assessing the perceived ChatGPT risks. Higher perceived risks were seen among females (*P*=.036, M-W; Table 5). No statistically significant differences were seen based on age, nationality, university, or self-reported ChatGPT use (Table 5).

For the technology/social influence subscale, the possible range was 5 to 25, with higher values indicating a positive attitude toward technology exemplified by ChatGPT and a score of 15 indicating a neutral attitude. The mean attitude toward technology score was 19.3 (SD 4.1), indicating a positive attitude toward ChatGPT technology. Higher technology subscale scores were seen among the participants who used ChatGPT before the study (mean 21, SD 3.6 vs mean 17.6, SD 3.9 among those who have not used it before the study; *P*<.001, M-W), and among males (mean 20.1, SD 4 vs mean 18.3, SD 4.2 among females; *P*=.023, M-W). No statistically significant differences were seen based on age, nationality, university, and educational level (Table 5).

For the anxiety subscale, the possible range was 3 to 15, with higher values indicating lower anxiety toward ChatGPT due to the reverse coding of these items and a score of 9 indicating a neutral attitude. The mean anxiety score was 6.6 (SD 2.9), indicating an anxious attitude regarding ChatGPT in the study sample. No statistically significant differences were seen based on age, sex, nationality, university, educational level, and self-reported ChatGPT use (Table 5).

**Table 5:** Comparison of the 3 TAME-ChatGPT^a^ attitude constructs stratified by participants’ variables.

Variables and constructs		Perceived risk				Technology/ social influence				Anxiety		
		Mean (SD)	Median (IQR)	*P* value	Mean (SD)		Median (IQR)	*P* value	Mean (SD)		Median (IQR)	*P* value
**Age (years)**				.61				.26				.26
	18-20	12.8 (5.5)	12.5 (9-16)		19.9 (4.1)		20 (16.5-24.5)		7.1 (3.4)		6 (4-10)	
	>20	12.3 (4.3)	12 (10-15)		19 (4.2)		19 (16-22)		6.2 (2.5)		6 (5-7)	
**Sex**				.04				.02				.24
	Male	13.3 (5.3)	13 (10-17)		20.1 (4)		20 (17-24)		6.9 (3.1)		6 (5-9)	
	Female	11.3 (3.8)	11 (9-14)		18.3 (4.2)		17.5 (15-21)		6.1 (2.7)		6 (4-7)	
**Nationality**				.62				.11				.43
	Jordanian	12.3 (4.4)	12 (10-15)		18.7 (4)		19 (15-22)		6.2 (2.4)		6 (5-7)	
	Non-Jordanian	12.7 (5.3)	12 (9-16)		20 (4.2)		20 (16-24)		6.9 (3.3)		6 (4-10)	
**University**				.57				.86				.82
	Public	12.3 (4.7)	12 (9-15)		19.3 (4.2)		19 (16-24)		6.6 (3)		6 (4-9)	
	Private	13.8 (5.7)	12 (11-14)		19.5 (3.6)		20 (19-21)		6.6 (2.6)		6 (6-7)	
**Educational level**				.96				.40				.52
	Undergraduate	12.5 (4.9)	12 (10-15)		19.4 (4.1)		20 (16-23.5)		6.7 (3)		6 (4-9)	
	Postgraduate	12.1 (3.9)	13 (10-15)		18.2 (4.5)		17 (15-21)		5.8 (2		6 (5-7)	
**Have you used ChatGPT before this study?**				.84				<.001				.353
	Yes	12.5 (5.2)	12 (9-17)		21 (3.6)		21 (18-25)		6.4 (2.9		6 (4-7)	
	No	12.4 (4.4)	12 (10-15)		17.6 (3.9)		17.5 (15-20)		6.8 (2.9		6 (5-9)	

^a^TAME-ChatGPT: Technology Acceptance Model Edited to Assess ChatGPT Adoption.

### Descriptive Analysis of ChatGPT Usage Determinants Based on TAME-ChatGPT

The 4 TAME-ChatGPT usage subscales were evaluated. The possible range of the perceived usefulness subscale was 6 to 30, with higher values indicating a higher perceived usefulness of ChatGPT and a score of 18 indicating a neutral attitude.

The mean perceived usefulness score was 24.2 (SD 4.9), indicating high perceived usefulness of ChatGPT among the participants who used it before the study. No statistically significant differences were seen based on age, sex, nationality, university, and educational level (Table 6).

For the perceived risk subscale, the possible range was 3 to 15, with higher values indicating lower perceived risks from ChatGPT use due to reverse coding of these items and a score of 9 indicating a neutral attitude. The mean perceived risk score was 7.2 (SD 2.8), indicating a slightly high perceived risk from ChatGPT use. No statistically significant differences were seen based on age, sex, nationality, university, and educational level (Table 6).

For the perceived ease of use subscale, the possible range was 2 to 10, indicating higher perceived ease of ChatGPT use, and a score of 6 indicated a neutral attitude. The mean perceived ease of use was 8.9 (SD 1.6), indicating the high perceived easiness of ChatGPT use in the study sample. No statistically significant differences were seen based on age, sex, nationality, university, and educational level (Table 6).

For the behavior subscale, the possible range was 3 to 15, with higher values indicating a positive behavior toward ChatGPT use due to reverse coding of these items and a score of 9 indicating a neutral attitude. The mean behavior was 9.8 (SD 3.3), indicating a slightly positive behavior toward ChatGPT leaning toward a neutral attitude. No statistically significant differences were seen based on age, sex, nationality, university, and educational level (Table 6).

**Table 6:** Comparison of the 4 TAME-ChatGPT^a^ usage constructs stratified by participants’ variables.

Variables and constructs		Perceived usefulness				Perceived risk				Perceived ease of use				Behavior		
		Mean (SD)	Median (IQR)	*P* value	Mean (SD)		Median (IQR)	*P* value	Mean (SD)		Median (IQR)	*P* value	Mean (SD)		Median (IQR)	*P* value
**Age**				.06				.78				.39	10 (3.7)		10 (8-14)	.58
	18-20	25.6 (5.1)	27(22-30)		7.4 (3.5)		7 (5-11)		9.2 (1.1)		10 (8-10)					
	>20	23.3 (4.6)	23(21-27)		7 (2.3		6.5 (6-8)		8.7 (1.8)		10 (8-10)		9.6 (3.2)		9 (8-12)	
**Sex**				.81				.14				.69				.51
	Male	24.1 (4.6)	24 (22-28)		7.5 (3)		7.5 (6-9)		9 (1.3)		10 (8-10)		10 (3.3)		10 (8-12)	
	Female	24.3 (5.5)	27 (21-29)		6.5 (2.5)		6 (5-7)		8.9 (2)		10 (8-10)		9.4 (3.5)		9 (7-12)	
**Nationality**				.16				.95				.45				.45
	Jordanian	23.4 (4.9)	23 (21-27)		7.2 (2.7)		6.5 (5-9)		9 (1.7)		10 (8-10)		9.4 (3.4)		9.5(6-12)	
	Non-Jordanian	25.1 (4.9)	26 (22-29)		7.1 (3)		7 (5-8)		8.8 (1.4)		10 (8-10)		10.2 (3.3)		10 (8-12)	
**University**				.91				.80				.52				.14
	Public	24.1 (4.9)	24 (21-28)		7.1 (3)		7 (5-9)		9 (1.6)		10 (8-10)		10 (3.2)		10 (8-12)	
	Private	24.7 (5.4)	27.5 (20-28)		7.2 (1.7)		7 (6-8)		8.5 (1.8)		9 (7-10)		7.8 (3.8)		6 (5-11)	
**Educational level**				.19				.65				.66				.91
	Undergraduate	24.4 (5)	24 (22-29)		7.2 (2.9)		7 (5-9)		9 (1.6)		10 (8-10)		9.7 (3.5)		10 (8-13)	
	Postgraduate	22.3 (3.1)	22 (21-24)		6.7 (2.3)		6 (6-7)		8.7 (1.6)		9 (8-10)		9.8 (2.6		10.5 (8-12)	

^a^TAME-ChatGPT: Technology Acceptance Model Edited to Assess ChatGPT Adoption.

## Discussion

### Principal Results

The main finding of this study demonstrated the reliability and validity of TAME-ChatGPT as a possible valuable tool for assessing health care students’ attitudes toward ChatGPT. The findings emphasized the need to account for risk perceptions, usefulness, ease of use, attitudes toward technology, and behavioral factors to successfully implement ChatGPT in health care education. These insights can guide AI developers, academics, and policy makers to formulate suitable strategies to ensure the ethical and optimal deployment of ChatGPT while addressing potential implementation challenges.

The availability of ChatGPT as an example of LLMs carries transformative societal implications, especially in health care settings, making its adoption in health care education seemingly inevitable [9,11,51-54]. Students will increasingly explore this innovative AI-based technology, with an already growing literature highlighting its significance in health care education through personalized learning with immediate feedback and impressive performance in medical exams [9,18,40,55-60]. Additionally, a recent study indicated a growing tendency among the general public to employ ChatGPT for self-diagnosis [61]. Therefore, the initial step toward the effective integration of ChatGPT in health care education involves evaluating attitudes toward this novel technology as well as the factors influencing its acceptance and usage.

However, before achieving this relevant aim, it is imperative to use a survey instrument that is validated to reach reliable conclusions based on the tested variables. Thus, this study represents one of the initial efforts to construct and validate a survey instrument assessing the attitudes toward ChatGPT among health care students in Jordan.

In this study, the major domains that were inferred through EFA included the perceived risks associated with ChatGPT, the attitude toward technology/social influence, and the anxiety that ChatGPT creates for the participants who have heard of ChatGPT. For the participants who used ChatGPT, EFA showed that 4 TAM-based domains were crucial factors driving ChatGPT use, which included the perceived usefulness, perceived risks, perceived ease of use, and behavior driving the use of technology.

The emergence of perceived risks as a major construct driving the attitude toward ChatGPT and its use is understandable. This is related to the potential for LLMs exemplified by ChatGPT to generate biased, inaccurate, or harmful content [9]. ChatGPT, among other LLMs, depends on huge training data sets; nevertheless, there is a general lack of transparency regarding the origin of these data [9,37]. Subsequently, there is a possibility that LLMs could learn and reproduce biased and incorrect content, which can have severe consequences in health care settings [9,36,37,62-64].

Risk perception plays a crucial role in decision-making, including the adoption of novel technologies like ChatGPT [65-68]. Recent studies highlighted the potential risks associated with ChatGPT risks including performance and privacy concerns [9,41]. Consequently, the participating students’ knowledge, beliefs, and prior experience with similar technologies significantly influenced their risk perception of ChatGPT. Unintended negative consequences, such as inappropriate or inaccurate content, pose significant risks in health care settings, necessitating careful consideration before its adoption in health care education [9,69-71].

This study demonstrated that risk perception significantly influenced health care students’ attitudes and usage of ChatGPT. This emphasizes the need for developers to address potential biases in ChatGPT, in addition to the need to address possible technological flaws to prevent cybersecurity threats and data breaches. Policy makers and AI-chatbot developers should prioritize transparent risk management strategies to promote responsible ChatGPT adoption in health care education [9,18,72]. Suggested measures to address ChatGPT’s perceived risks include student education on ChatGPT’s limitations and risks, establishing ethical guidelines for its responsible use, considering ethical and legal aspects, and promoting the development of high-quality training data [9,41].

The second construct driving the attitude toward ChatGPT found in this study was the attitude toward technology, alongside social influence. This construct refers to the perception and readiness to embrace technological innovations. Consistent with the previous evidence, positive attitudes facilitate the adoption of new technology adoption [73,74]. Thus, to promote a wider adoption of educational chatbots, providing training and education on the technology, highlighting its benefits, and ensuring accurate outputs are crucial [75,76].

Social influence can significantly impact attitudes toward ChatGPT adoption, including the opinions of the social circle and peers [77,78]. Additionally, media, public figures, and technology leaders play a role in shaping positive attitudes toward such applications. For example, the public opinions of prominent figures in the technology and business sectors can influence the widespread adoption and use of ChatGPT [79,80].

The third construct found in this validation study was the anxiety ChatGPT might provoke. The global availability of ChatGPT can be a transformative paradigm shift akin to the introduction of the internet and mobile phones, inducing fear, uncertainty, or discomfort [79,81,82]. Therefore, the elicited anxiety from such novel technology should be regarded as a significant factor driving its adoption [83,84].

In the second part of the TAM-based survey assessing ChatGPT usage determinants, the results showed that the perceived usefulness and ease of use as important factors influencing ChatGPT use among health care students. These psychological factors have been identified previously to play a critical role in shaping attitudes toward the adoption of new technologies [74,85-87]. Additionally, the perceived usefulness and effectiveness of technologies in achieving their intended goals could significantly influence the overall attitude of users, since an efficient and user-friendly technology encourages a more positive attitude toward its adoption [87-89]. Consequently, the impact of perceived usefulness and ease of use on students’ attitudes toward ChatGPT appear crucial for predicting and encouraging its successful adoption. In this exploratory study, we observed a high level of ease of use among the small group of participants who reported using ChatGPT, likely due to its user-friendly nature and free accessibility [17,71,90].

In this study, following the TAM model, the behavioral and cognitive factors emerged as key drivers of ChatGPT usage among health care students. ChatGPT can provide quick and easy access to information and services, reducing the need for human interaction, which is advantageous for busy health care students dealing with massive information and packed learning schedules [18,91]. Therefore, the ease of access provided by ChatGPT compared to traditional methods of education is a significant advantage [9,18,91,92]. Additionally, educational chatbots offer the potential to enhance self-confidence and communication skills, particularly for students facing challenges in social communication, highlighting its value as a conversational interface that simulates human interactions and fosters a sense of companionship among students [93,94].

On the other hand, one of the negative driving factors for ChatGPT use is the potential for dependence or even addiction [95]. This problem is of particular concern for individuals who may be susceptible to compulsive behavior [96]. This addiction can lead to decreased productivity, social withdrawal, and other negative consequences severely affecting the students’ later interactions with patients. The use of ChatGPT can also be associated with a deterioration in empathy and social skills [9]. The reliance on ChatGPT may result in hindering the development of the skills needed to interpret and respond to social cues, which should be considered in health care education [9,91].

### Limitations

The limited sample size used in this study is a major limitation; however, the complexity of the scale required the participants to spend considerable time and effort, which can limit the number of participants that are willing to complete the survey due to respondent fatigue [97]. Selection bias should also be considered based on the adoption of convenience-based sampling, and this issue should be addressed in future studies aiming to confirm the findings of this study and evaluate the attitudes of health care students toward ChatGPT and its use. The female predominance might be due to selection bias, but it aligns with the fact that dentistry, pharmacy, and nursing fields in Jordan have a majority of female students, as anticipated. Importantly, despite the utilization of the TAM framework, a significant limitation of this study is the potential bias in the tested constructs, which should be considered in future validation studies.

### Future Perspectives

Following the initial validation of TAME-ChatGPT as a tool to assess the attitude and usage of ChatGPT among health care students as indicated by the results of this study, a follow-up multinational project will ensue to conduct a confirmatory factor analysis and determine the major determinants of the attitude toward ChatGPT. This can help to guide the efforts needed for the successful adoption of ChatGPT in health care education.

### Conclusions

In this study, we showed that the validated TAME-ChatGPT scales have good reliability and validity with usefulness to test the following domains covered by 13 items to determine the attitude toward ChatGPT: perceived risks from ChatGPT, the attitude toward technology/social influence, and the anxiety that ChatGPT creates. Additionally, 4 constructs can be helpful to determine the factors driving ChatGPT use comprising 14 items: usefulness, perceived risks, perceived ease of use, and behavior driving the use of ChatGPT. Future studies are recommended to guide the successful adoption of ChatGPT in health care education.

Overall, the results of this study highlighted the importance of considering perceptions of risks, usefulness, ease of use, and attitudes toward technology as well as the behavioral factors upon adopting new technologies for health care education exemplified by ChatGPT. This can help AI developers, academics, and policy makers devise strategies to promote the effective and ethical use of ChatGPT and identify barriers to the adoption of this breakthrough revolutionary technology. By analyzing the acceptance and use of ChatGPT through a reliable and valid construct, evidence-based insights can inform decisions on the incorporation of this technology in health care education.

## Appendix

Multimedia Appendix 1 Supplementary tables and figures.

## Abbreviations

AI: artificial intelligence
EFA: exploratory factor analysis
GPT: Generative Pretrained Transformer
KMO: Kaiser-Meyer-Olkin
LLM: large language model
M-W: Mann-Whitney
PCA: principal component analysis
TAM: technology acceptance model
TAME-ChatGPT: Technology Acceptance Model Edited to Assess ChatGPT Adoption
UTAUT2: Unified Theory of Acceptance and Use of Technology 2
