# Supplementary material for: Assessing Health Students' Attitudes and Usage of ChatGPT in Jordan: Validation Study
Source: JMIR Med Educ. 2023 Sep 5;9:e48254. doi: 10.2196/48254 (PMC10509747; doi:10.2196/48254)
Supplement: Multimedia Appendix 1 [file mededu_v9i1e48254_app1.docx]

Sallam et al

**Assessing Attitudes and Usage of ChatGPT in Jordan Among Health Students: Validation Study of a Technology Acceptance Model–Based Scale (TAME-ChatGPT)**

**Multimedia Appendix 1**

**Table S1. The complete items evaluated to construct TAME-ChatGPT.**

| **Item** |
| --- |
| **Items tested among the respondents who heard of ChatGPT and among the respondents who used ChatGPT before the study** |
| 1. I am concerned about the reliability of the information provided by ChatGPT ^a^ |
| 2. I am concerned that using ChatGPT would get me accused of plagiarism ^a^ |
| 3. I fear relying too much on ChatGPT and not developing my critical thinking skills ^a^ |
| 4. I am concerned about the potential security risks of using ChatGPT ^a^ |
| 5. I am afraid of becoming too dependent on technology like ChatGPT ^a^ |
| 6. I am afraid that using ChatGPT would result in a lack of originality in my university assignments and duties ^a^ |
| 7. I am afraid that the use of the ChatGPT would be a violation of academic and university policies ^a^ |
| 8. I am concerned about the potential privacy risks that might be associated with using ChatGPT ^a^ |
| 9. I am enthusiastic about using technology such as ChatGPT for learning and research |
| 10. I believe technology such as ChatGPT is an important tool for academic success |
| 11. I think that technology like ChatGPT is attractive and fun to use |
| 12. I am always keen to learn about new technologies like ChatGPT |
| 13. I trust the opinions of my friends or colleagues about using ChatGPT |
| **Items tested among the respondents who used ChatGPT before the study** |
| 14. ChatGPT helps me to save time when searching for information |
| 15. For me, ChatGPT is a convenient method for accessing information |
| 16. For me, ChatGPT is a reliable source of accurate information |
| 17. ChatGPT helps me in better understanding of difficult topics and concepts |
| 18. ChatGPT makes it easier for me to complete the assignments in university courses |
| 19. I recommend ChatGPT to my colleagues to facilitate their academic duties |
| 20. ChatGPT is more useful than other sources of information that I have used previously |
| 21. I think that using ChatGPT has helped to improve my overall academic performance |
| 22. I have used tools or techniques similar to ChatGPT to in the past |
| 23. I spontaneously find myself using ChatGPT when I need information for my university assignments and duties |
| 24. I often use ChatGPT as a source of information in my university assignments and duties |
| 25. I appreciate the convenience and efficiency that ChatGPT provides for my university assignments and duties |
| 26. I think that relying on technology like ChatGPT can disrupt my critical thinking skills ^a^ |
| 27. I appreciate the accuracy and reliability of the information provided by ChatGPT |
| 28. I believe that using ChatGPT can save time and effort in my university assignments and duties |
| 29. I appreciate the importance of hands-on learning and experience, even if it means not relying on technology like ChatGPT ^a^ |
| 30. It does not take a long time to learn how to use ChatGPT |
| 31. ChatGPT is easy to use |
| 32. ChatGPT does not require extensive technical knowledge |
| 33. I do not face many difficulties when using ChatGPT |
| 34. The positive experiences of others have encouraged me to use ChatGPT |
| 35. I believe that people I know have improved their academic performance as a result of using ChatGPT |
| 36. I think using ChatGPT is important for me to keep up with my peers academically |

^a^ Items reversed coded.

**Figure S1. The scree plot of the ChatGPT attitude scale based on the principal component analysis.**


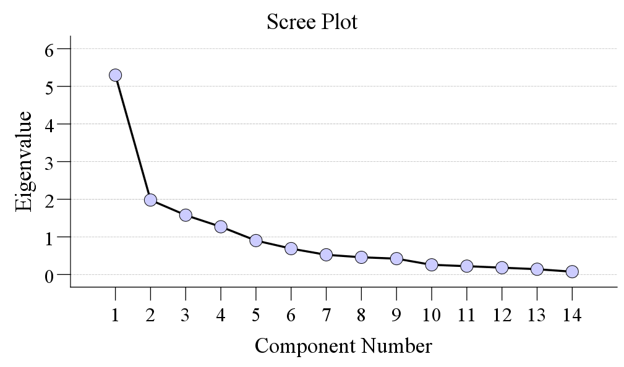


**Figure S2. The scree plot of the ChatGPT usage scale based on the principal component analysis.**

**
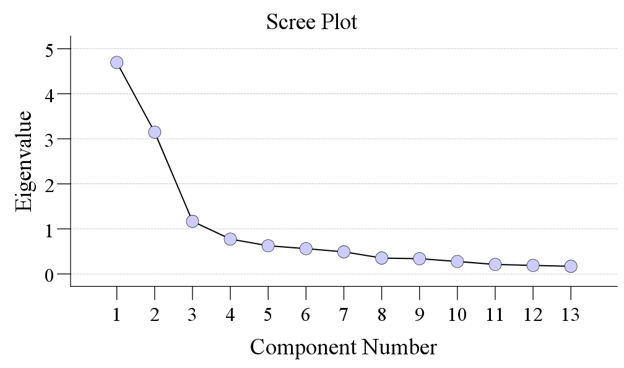
**
